# Exploring patient satisfaction with community pharmacy services in the United Arab Emirates: Implications for quality improvement

**DOI:** 10.1371/journal.pone.0339417

**Published:** 2026-01-30

**Authors:** Zelal Kharaba, Karem H. Alzoubi, Sayer Al-Azzam, Ahmad Al-Azayzih, Ahmad Y. Abuhelwa, Feras Jirjees, Anan Jarab, Azza Ramadan, Shoroq M. Altawalbeh, Dania Rahhal, Munazza Ahmed, Laila A. Ibrahim, Mamoon A. Aldeyab, Yassen Alfoteih, Sawsan Abuhammad

**Affiliations:** 1 Department of Pharmacy Practice and Pharmacotherapeutics, College of Pharmacy, University of Sharjah, Sharjah, United Arab Emirates; 2 Faculty of Medical Sciences, Newcastle University, Newcastle upon Tyne, United Kingdom; 3 Research Institute of Medical and Health Sciences, University of Sharjah, Sharjah, United Arab Emirates; 4 Department of Pharmaceutical Sciences, College of Pharmacy, QU-Health, Qatar University, Doha, Qatar; 5 Department of Clinical Pharmacy, Faculty of Pharmacy, Jordan University of Science and Technology, Irbid, Jordan; 6 Department of Pharmacology & Therapeutics, College of Medicine and Health Sciences, United Arab Emirates University, Al Ain, United Arab Emirates; 7 College of Pharmacy, Al Ain University, Abu-Dhabi, United Arab Emirates; 8 Department of Pharmacy, School of Applied Sciences, University of Huddersfield, Huddersfield, United Kingdom; 9 Reading School of Pharmacy, University of Reading, Reading, United Kingdom; 10 College of Dentistry, City University Ajman, Ajman, United Arab Emirates; 11 College of Humanities, City University Ajman, Ajman, United Arab Emirates; 12 Department of Nursing, College of Health Sciences, University of Sharjah, Sharjah, United Arab Emirates; 13 Department of Maternal Child Health and Midwifery, Faculty of Nursing, Jordan University of Science and Technology, Irbid, Jordan; University of South Australia, AUSTRALIA

## Abstract

**Introduction:**

Patient satisfaction is a critical metric for enhancing service quality, meeting regulatory standards, and validating patient-reported outcomes in healthcare. Community pharmacies play a vital role in healthcare delivery, yet there is limited research on patient satisfaction with these services in the UAE.

**Aims:**

This study aims to identify key factors influencing patient satisfaction with pharmaceutical care services provided by community pharmacies in the UAE.

**Methods:**

A cross-sectional, questionnaire-based study was conducted from December 1st, 2023, to April 30th, 2024. A systematic intercept sampling method was used to ensure a representative sample of 505 patients from various regions of the UAE. Data were collected through structured questionnaires covering demographic details, pharmacy visit experiences, and satisfaction levels. Statistical analyses, including chi-square tests, t-tests, and binary logistic regression, were performed using SPSS 27.

**Results:**

The study found that most participants most frequently used chain pharmacies (62.38%) and were highly satisfied with factors like lighting (91.29%) and pharmacist attentiveness (83.96%). Key drivers of satisfaction included convenient locations, accessible designs, and communication-related factors.. However, challenges such as the lack of private counseling areas (59.01%), limited access to medical files (77.62%), and inadequate prescription areas for private conversations (53.29%) were highlighted. Satisfaction was significantly lower in the Northern Emirates compared to Abu Dhabi, while differences involving Al Ain did not reach statistical significance. Providing sufficient time for medication advice (OR: 16.21, p < 0.001) and ease of waiting times (OR: 4.29, p = 0.016) improved satisfaction.

**Conclusion:**

The findings underscore the importance of both environmental and interpersonal factors in shaping patient satisfaction with community pharmacies. Enhancing pharmacy accessibility, communication, and the quality of pharmacist-patient interactions can significantly improve patient experiences. Future research should further explore overall satisfaction and investigate targeted improvements in pharmacy practices across different regions and settings.

## Introduction

Healthcare encompasses a range of services from preventive care and disease management to emergency response and specialized treatments, all aimed at promoting population well-being [1]. However, healthcare systems globally face significant challenges, including rising chronic disease rates that drive up healthcare expenditures [2]. These challenges are compounded by issues like staff behavior, responsiveness, and service affordability, leading to a decline in healthcare quality and accessibility for many communities [3].

Pharmacists, positioned at the most accessible point of the healthcare system, play a crucial role in bridging gaps in patient care [4,5]. Unlike other healthcare providers who may have limited availability or require appointments, pharmacists provide immediate support, expert advice, medication counseling, and preventive care. By assisting with medication management, delivering preventive services like vaccinations, and providing health education, pharmacists help alleviate healthcare system strains and improve patient outcomes by ensuring consistent care access.

In the United Arab Emirates (UAE), with a population of over 10 million [6], chronic diseases contribute significantly to morbidity and mortality [7]. Despite having over 12,000 pharmacists [8], community pharmacy services are often seen as product-focused, with minimal pharmaceutical care [9,10]. For instance, Kharaba et al. (2022) [1] and Alhomoud et al. (2016) [9] highlighted that community pharmacies in the UAE primarily focus on dispensing medications and selling health-related products, with limited emphasis on patient-centered services such as medication counseling and chronic disease management. Similarly, Jirjees et al. (2024) [10] noted that while community pharmacies in the UAE are accessible, their services are often transactional, with less focus on comprehensive pharmaceutical care. However, a systematic assessment of patient satisfaction—incorporating factors such as pharmacy environment, accessibility, communication quality, and the specific dynamics of pharmacist-patient interactions—remains underexplored.

Hence, structural strategies and health system reforms are needed to extend these roles. For example, a pharmacist-led, patient-centered approach to medication therapy has been shown to improve patient engagement with primary care providers [9]. Moreover, pharmacists’ active involvement can assist patients in selecting the most effective and safe self-care products, thereby further improving patient outcomes [11].

Healthcare authorities in the UAE place a strong emphasis on ensuring patient satisfaction across all healthcare sectors [12]. Understanding patient perceptions is critical as it directly influences patient engagement, trust, and outcomes. Patients who view healthcare providers positively are more likely to seek advice, adhere to treatment recommendations [13], and engage in preventive care [14], which is crucial for effective health management. By assessing patient satisfaction and understanding their perceptions, pharmacists can identify and address service quality gaps, making targeted improvements in communication, care delivery, and patient support, ultimately enhancing patient experiences and health outcomes. Given the limited research on patient satisfaction and attitudes toward community pharmacy services in the UAE, this study aims to fill this gap by evaluating factors influencing patient satisfaction. Specifically, it assesses patient satisfaction with the pharmaceutical care services provided by community pharmacists and explores consumer attitudes toward the available services in community pharmacies.

## Methodology

### Study design and sampling technique

This study employed a cross-sectional, exploratory design to identify and describe the key factors influencing patient satisfaction with community pharmacy services in the UAE. As this area of research is still developing in the UAE context, the primary aim was to generate insights and hypotheses regarding these relationships rather than to test a predefined theoretical model. Data were collected from December 1^st^, 2023, to April 30^th^, 2024. The target population consisted of patients who visited community pharmacies across different regions of the UAE. A systematic sampling method was employed in this study. In practice, we applied a systematic intercept technique, where every third eligible patient exiting the pharmacy was approached for participation. This approach was used across various community pharmacies in Abu Dhabi, Al Ain, Dubai, and the Northern Emirates. To strengthen the study, we recruited 505 participants, surpassing the recommended sample size of 384, which was calculated using the RaoSoft® calculator (RaoSoft, Inc. 2004, www.raosoft.com/samplesize.html). This larger sample size enhances the statistical power and generalizability of the findings.

Trained researchers administered the questionnaire to patients exiting community pharmacies. Participation in the study was voluntary, and all participants were fully informed about the study’s aims and assured of confidentiality before completing the survey. This study was reviewed by the Research and Ethics Committee at Al Ain University, which granted an exemption from ethical approval (approval number COP/AREC/19). Written informed consent was obtained from all participants before their involvement. To ensure confidentiality, all collected data were anonymized and securely stored, safeguarding participants’ privacy.

### Study tool, validation, and piloting

The questionnaire used in this study was adapted and modified from two previous studies: El-Sharif et al. 2017 [15] and Alhomoud et al. 2016 [9], which explored the quality and level of satisfaction with health services provided by pharmacists. The questionnaire was carefully structured with clear, accessible language to ensure it was suitable for the participants’ diverse educational and cultural backgrounds.

The questionnaire was developed in English and translated into Arabic through a rigorous forward and backward translation process by two bilingual researchers to guarantee its accuracy. The final version of the questionnaire was then assessed for clarity, relevance, and appropriateness by three university professors specialized in clinical pharmacy and pharmacy practice and three members of the public in the UAE. Each expert rated the items on a scale of 1–10 during a virtual session. The mean clarity, relevance, and appropriateness scores were 9.4, 8.3, and 9.1, respectively. Based on the panel’s feedback, necessary revisions were made.

A pilot study with 10 participants was conducted to test the reliability and comprehensibility of the questionnaire. Feedback from the participants was used to refine the survey and ensure that all questions were clear and understandable.

The survey was structured into several key domains to assess patient satisfaction with community pharmacy services comprehensively. The first domain collected demographic information, including participants’ age, gender, educational level, professional background (medical/ nonmedical), and frequency of pharmacy visits, as well as details about the type and location of the pharmacy. In our study, participants with a medical background were defined as those having formal education or professional experience in healthcare-related fields, such as medicine, pharmacy, nursing, dentistry, or allied health sciences. Those without such qualifications were categorized under “non-medical background”. The second domain focused on the pharmacy visit experience and assessed communication facilities, such as the prescription counter and counseling area, along with the pharmacy’s design, comfort, and accessibility to the pharmacist. It also evaluated the pharmacist’s visibility, access to patient records, cooperativeness, and the need for a third party due to language barriers. The third part explored the evaluation of community pharmacy services and focused on the pharmacist’s attention; time spent advising on medications, inquiry about health conditions, patient satisfaction with the provided information, and preferences for pharmacist gender and communication methods. The fourth part included information about reasons for pharmacy preferences, including proximity, ease of waiting times, and the pharmacist’s personality and knowledge, as well as services provided, such as blood pressure and glucose monitoring. In this part, the most frequently visited pharmacy type refers to the most frequently visited pharmacy rather than an explicit preference. Participants were asked which type of pharmacy (chain or independent) they typically visit for their medication and medical advice. This distinction is crucial, as frequent visits may be influenced by factors such as proximity, availability, or necessity, rather than an active preference for service quality. Since pharmacy visits were driven by frequency rather than explicit preference, we have ensured that our satisfaction analysis accounts for potential confounding factors (e.g., location, accessibility). The last part contained questions about patient education that focused on lifestyle advice, including physical exercise, healthy eating, smoking cessation, hypertension, diabetes, and contraceptives. The exclusion criteria comprised individuals under 18 or those not residing in the UAE. Participants who were not proficient in Arabic or English were also excluded, as they would not be able to comprehend or fully respond to the survey accurately. Furthermore, individuals who declined to participate or did not provide informed consent were excluded from the study.

Data were gathered using a structured questionnaire that included closed and open-ended questions. The primary outcome of interest was patient satisfaction with community pharmacy services, with a focus on the types of services utilized and the quality of care provided. The questionnaire also explored the effectiveness of pharmacist communication, the clarity of information provided during consultations, and the overall experience at the pharmacy.

Demographic factors such as age, gender, and educational level were considered potential predictors of satisfaction, while the frequency of pharmacy visits and prior experiences with pharmacy services were examined as potential confounders. By analyzing these variables, the study aimed to identify factors influencing patient satisfaction and provide insights for quality improvement in community pharmacy services.

It is worth mentioning that the evaluation of pharmacy experience and environment measured objective pharmacy-specific factors (e.g., accessibility, counseling areas, and pharmacy design), while satisfaction was assessed separately based on patient perceptions of service quality. In addition, satisfaction was treated as a binary variable by categorizing responses from multiple satisfaction-related questions. Participants who reported being “always” or “sometimes” satisfied across key survey items (e.g., pharmacist attentiveness, communication, and service quality) were classified as satisfied, while those reporting “rarely” or “never” satisfied” were classified as not satisfied. No single overall satisfaction score was used, and this approach allowed us to analyze predictors of satisfaction while ensuring consistency across different service aspects. This categorization aligns with methods used in previous patient satisfaction studies in pharmacy settings (e.g., Alhomoud et al., 2016; El-Sharif et al., 2017), where binary outcomes enabled clearer identification of satisfaction determinants.

Lastly, while a full psychometric validation (e.g., formal CVI, factor analysis) was beyond the scope of this study, the instrument’s validity was strengthened through its derivation from validated tools, expert review, pilot testing, and assessment of internal consistency. This multi-faceted approach is considered a robust method for ensuring tool appropriateness in applied health research contexts.

### Representativeness of the sample

The study employed a systematic intercept method to recruit 505 participants from various regions of the UAE, including Abu Dhabi, Al Ain, Dubai, and the Northern Emirates. The sample was diverse in terms of age, gender, nationality, and educational background, ensuring a broad representation of the general pharmacy-visiting population. Additionally, the calculated sample size exceeded the recommended threshold (384 participants, per the RaoSoft® calculator), enhancing the study’s statistical power and generalizability. The sample size was calculated with a 95% confidence level and a 5% margin of error

### Satisfaction measure

***Definition & Conceptual Framework***: Patient satisfaction was assessed as a multidimensional construct, including pharmacist communication, medication counseling, pharmacy accessibility, and service quality, that align with the core tenets of established service quality models, such as the tangibles, reliability, and assurance dimensions of SERVQUAL. While this study utilized a pragmatic approach adapted from prior pharmacy-specific tools [9,15], this conceptual alignment underscores the comprehensive nature of the satisfaction assessment.

***Survey Instrument & Scale***: Satisfaction was measured using Likert-scale items (e.g., 1 = Very Dissatisfied to 5 = Very Satisfied) adapted from validated instruments in previous studies (El-Sharif et al., 2017; Alhomoud et al., 2016).

***Validation & Reliability***: The questionnaire underwent expert validation (three pharmacy professors and three public representatives) and pilot testing (n = 10) to confirm clarity and appropriateness. Internal consistency of the satisfaction domains was assessed using Cronbach’s alpha. The calculated alpha for the satisfaction items was 0.88, which indicates good internal reliability. The satisfaction domain comprised items assessing pharmacist attentiveness, provision of advice, clarity of information, and pharmacy environment. These items were conceptually related, and their high alpha value supports their coherence as a single construct. The reliability testing was conducted post-pilot using the full dataset.

***Data Analysis***: Satisfaction scores were analyzed using descriptive statistics, chi-square tests, t-tests, and binary logistic regression to determine significant predictors of satisfaction.

***Potential Bias & Limitations***: We acknowledge response bias and ceiling effects as potential concerns and mitigated them by using structured questionnaires and ensuring diverse participant representation.

### Assessment of patient satisfaction

Patient satisfaction was assessed using multiple items covering key aspects of pharmacy services, including pharmacist attentiveness, consultation time, clarity of information, waiting times, and pharmacy accessibility. Perceived ease of waiting time was measured as a subjective assessment of whether the waiting period was considered reasonable and acceptable by the patient, rather than an objective measure of time duration. Responses were recorded on a 5-point Likert scale (1 = very dissatisfied to 5 = very satisfied). These items were adapted from validated tools (El-Sharif et al., 2017; Alhomoud et al., 2016) and refined to fit the UAE pharmacy context through expert review and pilot testing. Given ongoing discussions on satisfaction measurement, we acknowledge that while Likert-based assessments provide valuable insights, future studies may explore alternative models such as experience-based ratings or patient-reported outcome measures.

### Statistical analysis

The participants’ responses were encoded, and the data were analyzed using SPSS 27. Descriptive statistics were employed to summarize demographic characteristics, patterns of service use, and satisfaction levels. Inferential statistics, including chi-square tests and t-tests, were used to examine significant differences in satisfaction levels across various demographic groups, such as age, gender, and education level. Additionally, binary logistic regression was performed to analyze the relationship between satisfaction (as a binary outcome) and related factors, such as demographic variables and frequency of pharmacy visits, to determine the predictors of patient satisfaction.

Prior to performing the binary logistic regression, the Variance Inflation Factor (VIF) was calculated for all independent variables to assess potential multicollinearity. All VIF values were found to be below 3, well under the conservative threshold of 5, indicating that multicollinearity was not a substantive issue in the model.

## Results

### Sociodemographic characteristics

A total of 505 responses were collected and were valid for further analysis. Table 1 provides an overview of the sociodemographic characteristics of the participants. The distribution of participants’ age was well-balanced, ranging from 18 to more than 51 years. Females comprised the majority, accounting for 56.04% of the participants. Nearly half of the participants were single (49.9%), while 41.39% were married. The vast majority of the participants were Ex-pats Arab (74.85%), with other nationalities, including Ex-pats, non-Arabs, and locals, also represented. Two-thirds of the participants held a bachelor’s degree (67.33%) which is higher than the UAE national average of 26.6% for individuals with a bachelor’s degree or higher, as reported by the UAE National Bureau of Statistics in 2021 https://www.uaestatistics.gov.ae. Additionally, a significant proportion had a non-medical background (59.21%). Most participants were fluent in Arabic or English (91.09% and 80.79%, respectively). Moreover, approximately one-third of the participants reported having a chronic disease (32.87%).

**Table 1:** Sociodemographic characteristics of the enrolled participants.

Variables	N, Frequency (%)
**Age (years):**	
18–24 years	163 (32.28%)
25–32 years	106 (20.99%)
33–40 years	88 (17.43%)
41–50 years	88 (17.43%)
> 51 years	60 (11.88%)
**Sex:**	
Male	222 (43.96%)
Female	283 (56.04%)
**Marital status:**	
Married	209 (41.39%)
Single	252 (49.9%)
Divorced	28 (5.54%)
Widowed	16 (3.17%)
**Nationality:**	
Expats Arab	378 (74.85%)
Expats non-Arab	56 (11.09%)
Local	71 (14.06%)
**Level of education:**	
High school	96 (19.01%)
Bachelor	340 (67.33%)
Postgraduate (Master/ PhD)	69 (13.66%)
**Professional background:**	
Medical background	205 (40.59%)
Non-Medical background	299 (59.21%)
**Spoken language** **(you can select multiple answers):**	
Arabic	460 (91.09%)
English	408 (80.79%)
Urdu	16 (3.17%)
Other*	24 (4.95%)
**Do you have any chronic diseases?**	
Yes	166 (32.87%)
No	339 (67.13%)

* Bangla, Ukrainian, Kurdish, Tamil, German, Portuguese, Dutch, Turkish, French

### Pharmacists and pharmacies’ demographics

Table 2 provides detailed information about the demographics of pharmacists and pharmacies. Participants were asked about the type of pharmacy they most frequently visited, rather than their explicit preference. The majority of participants (62.38%) most frequently used chain pharmacies for their medication and medical advice. The participants dealt with pharmacy locations all across the UAE, with the highest concentration in Abu Dhabi (57.43%). While frequent visits may indicate preference, other factors such as convenience, location, or availability of services could influence this choice. It’s worth mentioning that future studies could further distinguish between actual preference and habitual visits to better assess their impact on satisfaction.

**Table 2:** Demographics of pharmacists and pharmacies.

Variable	N, Frequency (%)
**Pharmacy type most frequently visited for medication or advice**	
Chain pharmacy	315 (62.38%)
Independent pharmacy	190 (37.62%)
**Pharmacy location**	
Abu Dhabi	290 (57.43%)
Al Ain	59 (11.68%)
Dubai	46 (9.11%)
Northern Emirates*	110 (21.78%)
**Does the pharmacy have a high number of customers seeking medical advice daily?**	
Yes	343 (67.92%)
No	162 (32.08%)
**Number of customers seeking medical advice during the last pharmacy visit**	
No one, just me	61 (12.08%)
Less than 5 customers	213 (42.18%)
5–15 customers	144 (28.51%)
16–25 customers	26 (5.15%)
More than 25 customers	28 (5.54%)
Not sure/Do not know	33 (6.53%)
**Age of the pharmacist you most commonly interact with**	
20’s	65 (12.87%)
30’s	305 (60.4%)
40’s	97 (19.21%)
50’s	25 (4.95%)
More than 50’s	13 (2.57%)
**Frequency of pharmacy visits**	
Weekly	39 (7.72%)
Monthly	98 (19.41%)
Every three months	148 (29.31%)
Every six months	47 (9.31%)
Yearly	13 (2.57%)
When needed	160 (31.68%)
**Common reasons for visiting a pharmacy** (multiple selections allowed)	
Refill regular medications	172 (34.06%)
To collect a prescription medication	306 (60.59%)
Purchase over-the-counter (OTC) medications	279 (55.25%)
Seek advice and consultations from pharmacists	131 (25.94%)
Purchase personal care products (cosmetics, vitamins, contraceptives)	181 (35.84%)
Purchase medical devices (blood pressure monitors, blood glucose devices, pregnancy tests)	94 (18.61%)
Health screenings (e.g., blood pressure, blood glucose, pulse oximeter)	61 (12.08%)
Other reasons	0 (0%)
**If collecting a prescription medication, what was your experience?**	
Acquired it immediately	103 (33.66%)
Waited at the pharmacy	177 (57.84%)
Arrived later to pick it up	26 (8.5%)

*Northern Emirates includes Sharjah, Ajman, Fujairah, and Umm Al Quwain.

Regarding the daily number of customers seeking medical advice, 67.92% of participants reported that the number was high in their pharmacy. Additionally, 42.18% of participants noted that fewer than five customers were present during their last visit. Although the pharmacists with whom the participants interacted varied in their ages, the majority of them were in their thirties (60.4%).

Approximately one-third of participants visited the pharmacy as needed (31.68%). The primary reason for visiting the pharmacy was to collect prescription medication (60.59%), with 57.84% of participants spending some time waiting to receive their medication. Other significant reasons for visiting the pharmacy included seeking advice and consultation from the pharmacist (55.25%), purchasing personal care products (35.84%), and refilling regular medication (34.06%).

### Pharmacy visits experience and environment

Participants were asked about their experience in the last pharmacy visit (Table 3). High satisfaction rates were observed across different aspects of the pharmacy environment: appropriate counter separation (78.42%), appropriate light, visibility, and visual quality (91.29%), comfortable pharmacy design and decoration (85.15%), comfortable waiting area (70.69%), and absence of background noise (62.57%). Additionally, participants were satisfied with the pharmacists’ attention (83.96%), pharmacists’ cooperativeness and willingness to talk to the patients (89.5%), and language barrier absence (69.7%). However, more than half of the participants stated that there is no private counselling area (59.01%), 77.62% think that pharmacists have no access to their medical file and 53.29% stated that the prescription area was not helpful for private conversations.

**Table 3:** Pharmacy visit experience and environment.

Sentence	Yes (N, Frequency, %)	No (N, Frequency, %)
The prescription counter separating patients from the pharmacy personnel is appropriate for effective communication.	396 (78.42%)	109 (21.58%)
A private counseling area is available.	207 (40.99%)	298 (59.01%)
The pharmacy had appropriate lighting, visibility, and visual quality.	461 (91.29%)	44 (8.71%)
The pharmacy design and decoration were comfortable.	430 (85.15%)	75 (14.85%)
The waiting area was comfortable.	357 (70.69%)	148 (29.31%)
Accessing the pharmacist for a meaningful dialogue was easy.	404 (80%)	101 (20%)
The pharmacist was visible during your last visit	431 (85.35%)	74 (14.65%)
The pharmacist has access to your medical file	113 (22.38%)	392 (77.62%)
Getting the pharmacist’s attention was easy.	424 (83.96%)	81 (16.04%)
The pharmacist is cooperative and willing to talk to patients.	452 (89.5%)	53 (10.5%)
Due to a language barrier, there was a need to communicate with the pharmacist through a third party.	167 (33.07%)	338 (66.93%)
There was a significant amount of background noise or other distractions.	189 (37.43%)	316 (62.57%)
The pharmacy design was accessible for disabled patients, including features like wheelchair tracks and accessibility for elderly patients.	338 (66.93%)	167 (33.07%)
There was a language barrier in communication with the pharmacist.	153 (30.3%)	352 (69.7%)
The prescription area was suitable for a private conversation.	236 (46.73%)	269 (53.27%)

### Evaluation of community pharmacy services

Approximately one-third of the participants stated that sometimes the pharmacist asked the patient to provide information about their medications or health conditions (32.67%), while around 44% had rarely or never asked. The vast majority of the participants found the pharmacists friendly (95.05%), with 81.98% receiving sufficient time for medical advice. Satisfaction with the information received was notably high, with 42.18% always satisfied and 52.48% sometimes satisfied. About two-thirds of the participants preferred to talk with a pharmacist of the same gender (63.17%), and 60.5% were female. Notably, 66.34% prefer visiting the pharmacy to communicate with the pharmacist, while only 9.7% prefer communicating with the pharmacist via the phone (Table 4).

**Table 4:** Evaluation of Community Pharmacy Services.

Question	Response	N, Frequency (%)
**Did the pharmacist ask for information about your medications or health conditions?**	Never	91 (18.02%)
	Rarely	132 (26.14%)
	Sometimes	165 (32.67%)
	Often	73 (14.46%)
	Always	41 (8.12%)
**Are the pharmacists usually friendly?**	Yes, always	232 (45.94%)
	Yes, sometimes	248 (49.11%)
	No	25 (4.95%)
**Did the pharmacist give you enough time to advise you regarding your medication(s)?**	Yes	414 (81.98%)
	No	91 (18.02%)
**The amount of time the pharmacist spends with you**	Enough	386 (76.44%)
	Not Enough	119 (23.56%)
**On average, how much time does the pharmacist spend with you?**	Less than 5 minutes	155 (30.69%)
	5 minutes	225 (44.55%)
	10 minutes	95 (18.81%)
	More than 10 minutes	30 (5.94%)
**After visiting the pharmacy, did you feel satisfied with the information you received?**	Yes, always	213 (42.18%)
	Yes, sometimes	265 (52.48%)
	No	27 (5.35%)
**Do you prefer to talk with a pharmacist of the same gender?**	Yes	319 (63.17%)
	No	186 (36.83%)
**Do you order medication via mobile application?**	Yes	212 (41.98%)
	No	293 (58.02%)
**Do you prefer to communicate with the pharmacist by phone or in person?**	Phone	49 (9.7%)
	Visit to pharmacy	335 (66.34%)
	No preference	121 (23.96%)

### Reasons for pharmacy preferences

**Fig 1:**
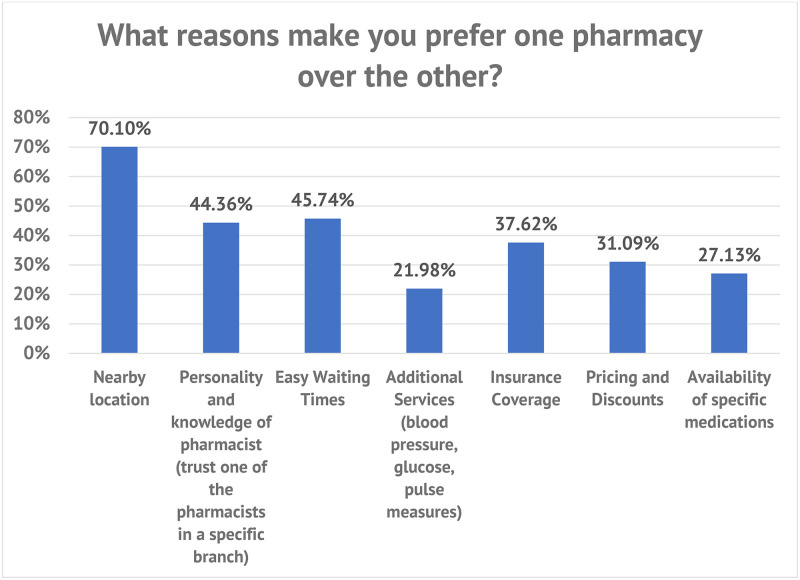
Reasons that make the patient prefer one pharmacy over the other.

The primary factor influencing customer preferences for one pharmacy over another was the proximity of the pharmacy, cited by 70.1% of participants. This was followed by ease of waiting times (45.74%) and the personality and knowledge of the pharmacist (44.36%). Conversely, providing additional services, such as blood pressure and glucose, was the lowest influencing factor (21.98%). Fig 1 provides more information.

### Patient education

Concerning patient education, less than half of the participants stated that they received lifestyle change advice from the pharmacist (44.16%). The most frequent advice the participants received was physical exercise and healthy eating (65.47% and 57.4%, respectively). Other advice was provided to the participants, such as smoking cessation, hypertension, diabetes, and contraceptives. Approximately half of the participants received education about the side effects of the medication (45.55%), while 51.68% were informed about the proper way of storage of medication. A significant proportion of pharmacists used written sources to explain drug therapy without a system that prints the therapy information (70.1%). Tables 5 and 6 show more details about patient education.

**Table 5:** Overview of Patient Education Provided by Pharmacists (n = 505).

Variables	Frequency (%)
**Have you ever received lifestyle change advice from a pharmacist?**	
Yes	223 (44.16%)
No	282 (55.84%)
**If yes, please select all that apply:**	
Smoking cessation	70 (31.39%)
Healthy eating	128 (57.4%)
Physical exercises	146 (65.47%)
Hypertension	44 (19.73%)
Diabetes	45 (20.18%)
Contraceptives	16 (7.17%)
Other (e.g., nutrition supplements)	2 (0.90%)

**Table 6:** Evaluation of Patient Education by the Pharmacist (n = 505).

Patient Education Items	Strongly Disagree (%)	Disagree (%)	Neutral (%)	Agree (%)	Strongly Agree (%)
The pharmacist clearly describes the side effects of the medication.	40 (7.92%)	90 (17.82%)	140 (27.72%)	179 (35.45%)	51 (10.1%)
If the pharmacy does not have a system to print the information, the pharmacist effectively explains the drug therapy using written sources.	11 (2.18%)	33 (6.53%)	102 (20.2%)	266 (52.67%)	88 (17.43%)
The pharmacist clearly explained the correct way to store the medication.	26 (5.15%)	61 (12.08%)	151 (29.9%)	206 (40.79%)	55 (10.89%)

### Binary regression analysis

The binary regression analysis in Table 7 explores the relationship between the satisfaction levels of the participants and different factors. Noteworthy results include the significant effect of pharmacy location on satisfaction. Participants in Northern Emirates reported significantly lower odds of satisfaction compared to those in Abu Dhabi (OR:0.14, 95% CI: 0.04–0.42, *p = 0.001*). Several factors were positively associated with higher satisfaction levels, including comfortable pharmacy design and decoration, accessibility of the pharmacy design for disabled patients, and perceived friendliness of pharmacists. Specifically, participants who found the pharmacy design comfortable and accessible for disabled patients had higher satisfaction (OR: 2.932, 95% CI: 0.99–8.64, *p = 0.048* and OR: 7.97, 95% CI: 2.47–30.92, *p = 0.001*, respectively). The presence of a language barrier also significantly increased satisfaction (OR: 5.83, 95% CI: 1.58–27.55, *p = 0.01*). Additionally, shorter waiting times were associated with improved satisfaction levels (OR: 4.29, 95% CI: 1.39–15.24, *p = 0.016*).

**Table 7:** A binary regression between satisfaction and related factors.

Characteristics		*p-value*	OR	95% C.I. for OR	
				Lower	Upper
Pharmacy location	Northern Emirates	0.001	0.14	0.04	0.42
	Alain	0.080	11.03	1.05	302.72
	Dubai	0.097	0.26	0.05	1.39
	Abu Dhabi	[Ref]	1.00	[Ref]	[Ref]
Comfortable Pharmacy design and decoration	Yes	0.048	2.93	0.99	8.64
	No	[Ref]	1.000	[Ref]	[Ref]
Was the pharmacy design accessible for disabled patients?	Yes	0.001	7.97	2.47	30.9
	No	[Ref]	1.00	[Ref]	[Ref]
Was there a language barrier in communication with the pharmacist?	Yes	0.014	5.83	1.58	27.55
	No	[Ref]	1.00	[Ref]	[Ref]
Are the pharmacists usually friendly?	Yes, all times	0.005	12.53	2.26	83.12
	Yes, sometimes	0.010	6.75	1.61	30.23
	No	[Ref]	1.00	[Ref]	[Ref]
Did the pharmacist give you enough time to advise you regarding your medication?	Yes	<0.001	16.21	5.33	59.29
	No	[Ref]	1.00	[Ref]	[Ref]
Easy Waiting Times	Yes	0.016	4.29	1.39	15.24
	No	[Ref]	1.00	[Ref]	[Ref]

Conversely, lower satisfaction was reported among participants who felt that they received insufficient time for medical advice (OR: 16.21, 95% CI: 5.33–59.29, *p=<0.001).*

## Discussion

Evaluating patient satisfaction is crucial for enhancing service quality, meeting regulatory standards, and validating patient-reported outcome measures. At the level of community pharmacies, such an evaluation can help practicing pharmacists pinpoint key areas for improving the quality in healthcare [16,17]. Additionally, it can support regulatory bodies in refining ministerial frameworks to enhance national healthcare policies, ultimately improving public health planning and outcomes. Thus, we aimed to identify the key factors influencing patient satisfaction with pharmaceutical care services by surveying patients visiting community pharmacies across the UAE. Patient satisfaction in this study was assessed through validated questionnaire items reflecting both direct contributors (e.g., pharmacist attentiveness, consultation time, and quality of information) and indirect facilitators (e.g., pharmacy accessibility, waiting times, and language barriers). While indirect factors shape overall experience, direct contributors have a more immediate impact on satisfaction.

Our results include patients with a diverse and well-balanced demographic profile, ages 18 to over 50 years, thus providing a broad range of perspectives across the generations. Most of the patients were female, and most were expatriate Arabs, though other nationalities, including expats, non-Arab, and locals, were also represented, reflecting the UAE’s inclusive and multicultural environment. Educationally, two-thirds held a bachelor’s degree, and most had a non-medical background, providing a balanced perspective with insights from both within and outside the healthcare field. Additionally, about one-third reported having a chronic disease, which highlights the prevalence of chronic conditions in the UAE [18]. Nonetheless, this diverse and educated sample strengthens the relevance and applicability of our findings.

This diversity is further reflected in participants’ pharmacy preferences, with the majority seeking their medications or medical advice from chain pharmacies rather than independent ones, likely due to perceptions of greater reliability and accessibility. However, Nind et al. found no substantial difference in the rate of medication adherence between users of independent and chain pharmacies [19], suggesting that perceived reliability does not necessarily translate to enhanced effectiveness of pharmacy services. Indeed, Rosalind Miller et al. investigated the performance of chain versus independent pharmacies in managing childhood diarrhea and suspected tuberculosis (TB) in urban India and found no significant differences in the management of suspected TB [20]. Elsewhere, Rosalind Miller et al. also reports that chain and independent pharmacies in India show minimal differences in staff qualifications, regulation, and self-regulation, with chain pharmacies prioritizing customer service over quality of care [21]. These findings suggest that since both pharmacy types offer comparable care, the key to proper management may lie in enhancing overall pharmacy management practices, mainly through targeted education and training to improve the quality of care delivered.

Furthermore, most respondents report that the pharmacies they visit handle many customers seeking medical advice daily, highlighting their critical role as health consultation centers beyond just medication dispensing. This highlights the importance of enhancing patient satisfaction with pharmacist advice, as studies suggest that doing so can lead to both financial benefits and health improvements for pharmacists and patients alike [22,23]. Moreover, patients are more likely to be satisfied and willing to pay for counseling if they receive adequate knowledge and find the pharmacist helpful [24].

Similarly, our respondents’ visits to pharmacies are primarily driven by immediate needs, such as collecting prescription medications, purchasing OTC medications, and refilling regular prescriptions rather than scheduled interactions. This further emphasizes the critical role of community pharmacists who can offer reliable advice and support to patients and act as accessible healthcare providers who can fulfill both immediate healthcare needs and offer preventive care through professional counseling.

Nevertheless, more than half of the respondents had to wait at the pharmacy before collecting their prescriptions. This contrasts with a nationwide study conducted previously by El-Sharif et al. in 2017, where 50.5% (n = 375) of the respondents could collect their prescriptions ‘right away.’ [15]. Such an increase in waiting time in pharmacies can be associated with factors such as higher patient volume, staff workload, and prescriptions requiring interventions, which, while improving safety, also lengthen processing times [25]. To combat this, Sadi et al. examined the utilization of electronic prescriptions in a public hospital pharmacy in the UAE and reported a reduction in patient waiting times of around 80%, which ultimately resulted in 82% of patients being extremely satisfied or satisfied with the pharmacy’s services [26]. Thus, as a recommendation, incorporating technology in community settings can reduce medication administration time and medication errors. Additionally, workforce disruptions and increased service demands may have affected pharmacies’ ability to serve customers promptly..

The high satisfaction rates with pharmacists’ attentiveness, cooperativeness, and willingness to engage suggest that patients value approachable, attentive, and communicative pharmacists. This reflects a positive perception of pharmacists as accessible healthcare providers contributing significantly to the patient experience. Such positive perceptions can also be seen from previous studies conducted in the UAE with high satisfaction rates related to their attentiveness, cooperativeness, and communication skills [9,27,28]. Moreover, a significant proportion of participants reported satisfaction despite the presence language barriers, indicating that communication-related factors remain closely linked to patient perceptions of pharmacy services.

More importantly, our binary regression analysis identified several key factors associated with patient satisfaction in community pharmacies. Pharmacy location showed a significant association with satisfaction only for participants from the Northern Emirates, who reported significantly lower satisfaction compared to Abu Dhabi, while the associations observed for Al Ain and Dubai did not reach statistical significance. These results suggest that geographical disparities—particularly between Abu Dhabi and the Northern Emirates—may influence patient experience. Rogers et al. reported that regional disparities could be influenced by the inverse care law [29]; however, further investigation is needed to clarify these patterns in the UAE context.[30]. Additionally, pharmacies that were accessible to disabled patients showed a strong positive association with satisfaction, emphasizing the importance of inclusive design in enhancing patient experiences. Interestingly, the presence of a language barrier was also significantly associated with higher reported satisfaction, suggesting that other factors—such as pharmacist attitude, time spent with patients, or interpersonal skills—may mitigate the negative impact of communication challenges [31,32].

The finding that nearly two-thirds of participants preferred a pharmacist of the same gender likely reflects the sociocultural and religious norms prevalent in the region, where modesty and gender concordance in healthcare interactions are highly valued. This has direct implications for pharmacy management in staffing and scheduling to accommodate patient preferences and improve comfort levels. Furthermore, the critical role of clear communication is underscored by the strong associations observed for pharmacist friendliness and the time allocated for patient counseling. These interpersonal factors may help explain why satisfaction remained high even when language barriers were reported, particularly in a nation with a predominantly expatriate population. Ensuring pharmacists are not only linguistically capable but also patient-centered and culturally competent remains essential for sustaining high-quality pharmacy services and building patient trust. The regression model identified key predictors of satisfaction, with pharmacist attentiveness (OR: 16.21, p < 0.001) and pharmacy accessibility (OR: 7.97, p = 0.001) showing strong associations. Easy waiting times were also significantly associated with higher satisfaction (OR: 4.29, 95% CI: 1.39–15.24, p = 0.016).The interaction between pharmacists and patients was identified as a pivotal determinant of patient satisfaction, particularly when pharmacists provided sufficient time for medication counseling. Remarkably, the perception that pharmacists consistently provided sufficient time to advise on medication was strongly associated with higher levels of satisfaction, as evidenced by an odds ratio (OR) of 16.21 (95% CI: 5.33–59.29, (*p < 0.001*)). Additionally, perceived ease of waiting times emerged as another significant factor influencing satisfaction, with patients reporting easy waiting times being significantly more likely to be satisfied (OR: 4.29, 95% CI: 1.39–15.24, p = 0.016)These findings highlight the multifaceted nature of patient satisfaction in pharmaceutical services, emphasizing the combined importance of operational efficiency and meaningful pharmacist-patient interactions. These findings are consistent with the satisfaction trends observed in hospital pharmacies [26], where quick service often correlates with higher satisfaction levels, highlighting potential similarities in patient expectations between community and hospital settings.

Efforts to improve pharmacy accessibility, communication, and the quality of pharmacist-patient interactions may significantly enhance patient experiences. Finally, the finding that fewer than half of respondents received lifestyle advice is concerning, given the UAE’s high burden of chronic diseases such as diabetes, cardiovascular diseases, and obesity. Lifestyle modifications play a critical role in preventing and managing these conditions, and the limited provision of such counseling in community pharmacies may represent a missed opportunity for public health intervention. Strengthening pharmacist-led lifestyle counseling initiatives could enhance chronic disease management and support national health strategies aimed at reducing non-communicable disease prevalence.

## Conclusion

Our findings show that where a pharmacy is located, how attentive and approachable the pharmacist is, and whether they take the time to properly counsel patients are all vital to patient satisfaction in the UAE. To achieve meaningful improvements, coordinated efforts from all stakeholders in pharmacy care are required. We recommend that health authorities update licensing rules to ensure pharmacies have private areas for conversations and are fully accessible to people with disabilities. They should also support better systems that let pharmacists securely access relevant patient health information. For pharmacy owners and managers, investing in continuous professional development —especially in communication skills, cultural understanding, and lifestyle counselling for chronic diseases management is essential. The adoption of technologies such as electronic prescribing may further support efficient service delivery by reducing waiting times while maintaining high standards of care. Finally, pharmacists themselves can make a major difference by consciously dedicating time for personal consultations and being aware that many patients feel more comfortable speaking with a pharmacist of the same gender. We encourage future studies to put these recommendations into practice and measure how they affect patient satisfaction and health across different emirates.

### Limitations of the study

The reliance on self-reported data may introduce response bias, as participants might misestimate their satisfactions due to recall errors or social desirability. Additionally, the questionnaire did not assess overall satisfaction as a single outcome, limiting our ability to measure the cumulative impact of all identified factors. Future research should adopt longitudinal designs to capture satisfaction trends more comprehensively.

Another limitation of this study is the relatively small sample size for Al Ain (n = 59), which may have contributed to the wide confidence interval observed for this region’s odds ratio. Data sparsity can impact the stability of the odds ratio estimates, potentially affecting the reliability of the results for this specific location. A potential other limitation is the exclusion of non-proficient Arabic/ English speakers, which may introduce selection bias and limit the generalizability of our findings, particularly for expatriate communities who primarily communicate in other languages. Another limitation is that dichotomizing multi-item Likert-scale satisfaction responses into a binary outcome may have limited the depth of analysis by reducing variability and statistical power. Although this approach improved model interpretability, future research should consider advanced methods like ordinal logistic regression to better capture satisfaction nuances. In addition, although we aimed for geographic representativeness, the use of systematic sampling rather than true random sampling may have introduced selection bias. This non-probabilistic approach limits the generalizability of our findings. Another limitation of the study that a potential interaction effects and statistical assessments of confounding were not explored due to sample size and model complexity constraints. Future research should incorporate advanced multivariable techniques to assess effect modification and control for confounders more rigorously. Finally, this cross-sectional study captures satisfaction at a single time point, and interactions between factors were not assessed. Longitudinal studies and mediation analyses are recommended to better understand the complex relationships influencing patient satisfaction.

## Supporting information

S1 File The Patient Satisfaction questionnaire. (PDF)

S2 File The study data set. (XLSX)
