# Supplementary material for: Exploring patient satisfaction with community pharmacy services in the United Arab Emirates: Implications for quality improvement
Source: PLoS One. 2026 Jan 30;21(1):e0339417. doi: 10.1371/journal.pone.0339417 (PMC12857972; doi:10.1371/journal.pone.0339417)
Supplement: S1 File — (PDF) [file pone.0339417.s001.pdf]

<u>Satisfaction questionnaire</u>

Section 1. Sociodemographic characteristics of the enrolled participants.

1. **Age (years):**
   - ☐ 18-24 years
   - ☐ 25-32 years
   - ☐ 33-40 years
   - ☐ 41-50 years
   - ☐ more than 50 years
2. **Sex:**
   - ☐ Male
   - ☐ Female
3. **Marital status:**
   - ☐ Married
   - ☐ Single
   - ☐ Divorced
   - ☐ Widowed
4. **Nationality:**
   - ☐ Expats Arab
   - ☐ Expats non-Arab
   - ☐ Local
5. **Level of education:**
   - ☐ High school
   - ☐ Bachelor
   - ☐ Postgraduate (Master / PhD)
6. **Professional background:**
   - ☐ Medical background
   - ☐ Non-Medical background

7. **Spoken language (you can select multiple answers):**
    - ☐ Arabic
    - ☐ English
    - ☐ Urdu
    - ☐ Other
8. **Do you have any chronic diseases?**
    - ☐ Yes
    - ☐ No
9. **What is the type of pharmacy you seek to get your medication or seek medical advice MOST OF TIMES?**
    - ☐ Chain pharmacy
    - ☐ Independent Pharmacy
10. **Pharmacy location:**
    - ☐ Abu Dhabi
    - ☐ Alain
    - ☐ Dubai
    - ☐ Northern Emirates (Sharjah, Ajman, Fujairah, Umm Al Quwain)
11. **Based on the type of pharmacy that is visited most often, does the pharmacy have a high number of customers seeking medical advice on a daily basis?**
    - ☐ Yes
    - ☐ No
12. **During the time you spent in the pharmacy at your last visits, how many customers were there to seek their medical advice from the same pharmacy?**
    - ☐ No one, just me
    - ☐ Less than 5 customers
    - ☐ 5 - 15 customers
    - ☐ 16-25 customers

- ☐ More than 25 customers
- ☐ Not sure/Don't know

13. Age of Pharmacist that you in general interact with?

- ☐ 20's
- ☐ 30's
- ☐ 40's
- ☐ 50's
- ☐ More than 50's

14. What are <u>the most common reasons</u> that lead you to visit a pharmacy? (you can select multiple answers)

- ☐ Refill regular medications
- ☐ To collect a prescription medication
- ☐ Purchase over-the-counter (OTC) medications
- ☐ Seek advice and consultations from pharmacists
- ☐ Purchase Personal care products (cosmetics, vitamins and minerals, contraceptives…)
- ☐ Purchase a medical device (blood pressure monitoring device, blood sugar device, pregnancy kit...)
- ☐ Health screenings: such as blood pressure checks, blood glucose monitoring, pulse oximeter
- ☐ Other reasons

15. If you choose (to collect a prescription medication):

- ☐ I acquired it right away.
- ☐ I spent some time waiting at the pharmacy.
- ☐ I arrived later to pick it up.

16. How many times do you visit the pharmacy?

- ☐ Weekly
- ☐ Monthly
- ☐ Every three months

- ☐ Every six months
- ☐ Yearly
- ☐ When I need something

Section 2: In the last time you visited a pharmacy, please answer the following questions regarding the last pharmacy you visited:

17. **The prescription counter separating patients from the pharmacy personnel is appropriate for effective communication.**
    - ☐ Yes
    - ☐ No

18. **Was there a private area (counseling area)?**
    - ☐ Yes
    - ☐ No

19. **Appropriate light, visibility, and visual quality were there.**
    - ☐ Yes
    - ☐ No

20. **Comfortable Pharmacy design and decoration were there.**
    - ☐ Yes
    - ☐ No

21. **The waiting area was comfortable.**
    - ☐ Yes
    - ☐ No

22. **It was easy for me to access the pharmacist to have a meaningful dialogue.**
    - ☐ Yes
    - ☐ No

23. **Was the pharmacist visible to you during your last visit?**
    - ☐ Yes
    - ☐ No

24. **Was the pharmacist have access to your medical file?**

- ☐ Yes
- ☐ No

25. Was it easy to get the pharmacist's attention on your last visit?
- ☐ Yes
- ☐ No

26. **In your opinion, do you think that the pharmacist is cooperative and willing to talk to patients**?
- ☐ Yes
- ☐ No

27. Do you have to speak to the pharmacist through a third party (for example: the pharmacist's dialect is not understandable and there is a need for another person to explain)?
- ☐ Yes
- ☐ No

28. Was there a lot of background noise or other distractions?
- ☐ Yes
- ☐ No

29. In your opinion, Was the pharmacy design accessible for disabled patients? For example, is there any wheelchair-specific track, easy to access by special groups such as elderly patients with walking difficulties?
- ☐ Yes
- ☐ No

30. Was there a language barrier in communication with the pharmacist?
- ☐ Yes
- ☐ No

31. The prescription area helpful for a private conversation
- ☐ Yes
- ☐ No

Section 3: Evaluation of the community pharmacy services

32. The pharmacist asked me to provide information about my medications or health conditions:

- ☐ Never
- ☐ Rarely
- ☐ Sometimes
- ☐ Often
- ☐ Always

33. **Are the pharmacists usually friendly?**

- ☐ Yes, all times
- ☐ Yes, sometimes
- ☐ No

34. Did the pharmacist give you enough time to advise you regarding your medication(s) (for example: giving you attention)?

- ☐ Yes
- ☐ No

35. **The amount of time the pharmacist spends with you:**

- ☐ Enough
- ☐ Not Enough

36. **On average time:**

- ☐ Less than 5 mins
- ☐ 5 mins
- ☐ 10 mins
- ☐ More than 10 mins

37. After visiting the pharmacy, did you feel satisfied with the information you were receiving?

- ☐ Yes, all times
- ☐ Yes, sometimes
- ☐ No

38. **What reasons make you prefer one pharmacy over the other? Tick all that applied.**

- ☐ Nearby location

- ☐ Personality and knowledge of pharmacist (trust one of the pharmacists in an specific branch)
- ☐ Easy Waiting Times
- ☐ Additional Services (blood pressure, glucose, pulse measures)
- ☐ Insurance Coverage
- ☐ Pricing and Discounts
- ☐ Availability of specific medications

39. **Do you prefer to talk with a pharmacist from the same gender?**
    - ☐ Yes
    - ☐ No

40. **Do you order medication via mobile application?**
    - ☐ Yes
    - ☐ No

41. **Do you prefer to communicate with the pharmacist by phone or visit the pharmacy?**
    - ☐ Phone
    - ☐ Visit to pharmacy
    - ☐ No difference

Section 4. Patient education by the pharmacist:

42. **Have you ever been given lifestyle change advice from a pharmacist?**
    - ☐ Yes
    - ☐ No

43. **If yes, choose multiple answers:**
    - ☐ Smoking cessation
    - ☐ Healthy eating
    - ☐ Physical exercises
    - ☐ Hypertension

- ☐ Diabetes
- ☐ Contraceptives
- ☐ Other

Section 5. Patient education by the pharmacist:

44. The side effects are clearly described by the pharmacist.

- ☐ Strongly disagree
- ☐ Disagree
- ☐ Neutral
- ☐ Agree
- ☐ Strongly agree

45. If the pharmacy, you visited doesn’t have a system to print the information does the pharmacist effectively explain the drug therapy using written sources:

- ☐ Strongly disagree
- ☐ Disagree
- ☐ Neutral
- ☐ Agree
- ☐ Strongly agree

46. Does the pharmacist inform you about the correct way to store the medication:

- ☐ Strongly disagree
- ☐ Disagree
- ☐ Neutral
- ☐ Agree
- ☐ Strongly agree
